# Effects of bench press technique variations on musculoskeletal shoulder loads and potential injury risk

**DOI:** 10.3389/fphys.2024.1393235

**Published:** 2024-06-21

**Authors:** L. Noteboom, I. Belli, M. J. M. Hoozemans, A. Seth, H. E. J. Veeger, F. C. T. Van Der Helm

**Affiliations:** ^1^ Faculty of Behavioral and Movement Sciences, Department of Human Movement Sciences, Amsterdam Movement Sciences, Vrije Universiteit Amsterdam, Amsterdam, Netherlands; ^2^ Department of Biomechanical Engineering, Delft University of Technology, Delft, Netherlands; ^3^ Department of Cognitive Robotics, Delft University of Technology, Delft, Netherlands

**Keywords:** injury prevention, biomechanics, musculoskeletal model, shoulder, rotator cuff, glenohumeral joint, strength training, bench press

## Abstract

While shoulder injuries resulting from the bench press exercise are commonly reported, no biomechanical evidence for lowering injury risk is currently available. Therefore, the aim of the present study was to compare musculoskeletal shoulder loads and potential injury risk during several bench press variations. Ten experienced strength athletes performed 21 technical variations of the barbell bench press, including variations in grip width of 1,1.5 and 2 bi-acromial widths (BAW), shoulder abduction angles of 45°, 70° and 90°, and scapula poses including neutral, retracted, and released conditions. Motions and forces were recorded by an opto-electronic measurement system and an instrumented barbell. An OpenSim musculoskeletal shoulder model was employed to estimate joint reaction forces in the glenohumeral and acromioclavicular joints. Time-series of joint reaction forces were compared between techniques by statistical non-parametric mapping. Results showed that narrower grip widths of <1.5 BAW decreased acromioclavicular compression (*p* < 0.05), which may decrease the risk for distal clavicular osteolysis. Moreover, scapula retraction, as well as a grip width of <1.5 BAW (*p* < 0.05), decreased glenohumeral posterior shear force components and rotator cuff activity and may decrease the risk for glenohumeral instability and rotator cuff injuries. Furthermore, results showed that mediolaterally exerted barbell force components varied considerably between athletes and largely affected shoulder reaction forces. It can be concluded that the grip width, scapula pose and mediolateral exerted barbell forces during the bench press influence musculoskeletal shoulder loads and the potential injury risk. Results of this study can contribute to safer bench press training guidelines.

## 1 Introduction

The barbell bench press is one of the most popular exercises in the gym. It is considered a benchmark exercise for upper body strength and plays an important role for recreational and competitive strength athletes and powerlifters (ACSM, 2012). It is therefore not a surprise that a large body of scientific work concerning the bench press exercise is available. This work typically involves investigations of the bench press technique leading to the best performance, focusing mainly on performance-related outcomes like power production, the one or six repetition max (1RM or 6RM), and muscle activation patterns (Wagner et al., 1992; Saeterbakken et al., 2017; Larsen et al., 2021; Martínez-Cava et al., 2022; López-Vivancos et al., 2023). Overall, most studies agree that the highest 1RM or 6RM and highest pectoralis major activation, which are common performance goals, can be achieved for a bench press technique with wide grip widths of two bi-acromial widths (BAWs), large shoulder abduction angles approaching 90°, and a full range-of-motion (ROM) (Wagner et al., 1992; Saeterbakken et al., 2017; Larsen et al., 2021; Martínez-Cava et al., 2022; López-Vivancos et al., 2023).

Unfortunately, musculoskeletal pain and injuries associated with the bench press exercise are a common problem among elite and recreational lifters, especially at the shoulder complex (Bengtsson et al., 2018). Specific bench press-related injuries that have been reported include Distal Clavicular Osteolysis (DCO), pectoralis major rupture, glenohumeral (GH) instability and rotator cuff injury (Durall et al., 2001; Green and Comfort, 2007; Bengtsson et al., 2018). Although high quality data regarding specific bench press injuries are lacking, one study estimated the prevalence of DCO in competitive weightlifters to be 27% (Scavenius and Iversen, 1992). Bench pressing at high intensity or frequency has been identified as risk factor for DCO (Nevalainen et al., 2016). Furthermore, although pectoralis major ruptures were previously characterized in the literature as rare injuries, Hauschild et al. (2021) showed that when overuse and mild to moderate injuries are included, pectoralis major injuries, almost all caused by bench pressing, form an underestimated and increasing problem in the military population. These pectoralis major injuries have an incidence of 6 injuries per 1,000 soldiers per year. Another study examined strength training participants that reported self-assessed shoulder pain due to bench pressing, and found that 76% appeared to have tendinitis, from which 56% had rotator cuff tendinitis and 20% had biceps tendinitis (Sharma and Singh Vij, 2005).

Despite the reported injuries, there appears to be a lack of understanding about bench press technique as a risk factor for injury. The available literature mainly consists of clinical expert opinions (Fees et al., 1998; Durall et al., 2001; Green and Comfort, 2007), which provide theories of injury mechanisms without biomechanical evidence. Multiple theories regarding potential bench press injury mechanisms have been developed, for instance, clinical experts argue that shoulder abduction angles larger than 45° and wide grips of 2 bi-acromial widths during the bench press could theoretically lead to high compression forces in the acromioclavicular (AC) joint (Fees et al., 1998; Green and Comfort, 2007). It is hypothesized that these high forces, especially when applied repetitive, may lead to microtrauma at the subchondral bone of the distal clavicular head, which may increase the risk for DCO (Fees et al., 1998; Green and Comfort, 2007; Schwarzkopf et al., 2008). Moreover, it is theorized based on anatomical knowledge that bench pressing with wide grips and large shoulder abduction angles approaching 90° could reduce the subacromial space and impinge the rotator cuff and could lead to high strain on the inferior glenohumeral ligament and anterior glenohumeral ligament that are responsible for anterior GH stability (Durall et al., 2001; Green and Comfort, 2007). In some cases, theories regarding injury mechanisms are contradictory. For instance, the National Strength and Conditioning Association (NSCA) recommends continuous retraction of the scapulae to provide a stable base of support (Graham, 2003). In contrast, based on clinical experience, Larsen (2008) proposed that a more natural re- and protraction rhythm during the bench press could help to maintain the humeral head on the glenoid fossa and suggested to manipulate this rhythm by placing a swimming pool noodle beneath the spine of the athlete to release the scapulae.

While there are several theories regarding the potential bench press injury mechanisms, there is currently a lack of biomechanical evidence to substantiate these theories. Most biomechanical studies regarding the bench press only used electromyography (EMG) measurements (López-Vivancos et al., 2023) and few calculated shoulder moments additionally (Larsen et al., 2021; Mausehund et al., 2022). Although these measures provide some indication of the risk, more detailed information is necessary. Especially for the shoulder girdle complex, which consists of multiple joints, information regarding reaction forces in the separate joints is necessary to link loading with specific bench press injuries. In particular, AC compression forces may be linked to DCO and GH reaction forces and rotator cuff activities may be linked to glenohumeral instability and rotator cuff injuries. These data are currently lacking in the literature. One way to gain more insight in muscle and joint loading is the use of a detailed musculoskeletal model in combination with kinematic and kinetic data from athletes performing the bench press.

Therefore, the objective of the present study is to investigate the effects of bench press technique variations in grip width, shoulder abduction angle, and scapula pose on the magnitude and direction of reaction forces at the glenohumeral and acromioclavicular joints, by combining athlete recordings during multiple technique variations with musculoskeletal shoulder model simulations. The present study will provide biomechanical evidence that may be used to specify safer bench press training guidelines.

## 2 Methods

### 2.1 Experimental approach to the problem

The present study has an experimental design. Multiple aspects of the bench press technique are manipulated to investigate the effect of bench press technique on musculoskeletal shoulder loads.

### 2.2 Subjects

Ten healthy, experienced strength athletes (sex: 9 male 1 female, age: 27 ± 3 years, bench press experience: 6.7 ± 3.9 years, body height: 1.80 ± 0.10 m, body mass: 87 ± 8 kg; mean ± standard deviation (SD)) were included in the present study. The inclusion criteria were an age of 18+ years, a minimum of 3 years experience in bench pressing, and no musculoskeletal injuries at the start of the study. The study was approved by the local ethics committee of the Delft University of Technology (ID:1648). All participants provided written informed consent after being informed on the aims and procedures of the experiment.

### 2.3 Procedures

Measurements of the participant’s body height, body mass, and bi-acromial distance were taken during preparation. Subsequently, 14 reflective markers were attached to the bony landmarks (Supplementary Table S1). Since the scapula mainly moves beneath the skin, it is not possible to track its motion by attaching markers on the scapula’s bony landmarks. Instead, a cluster marker was placed on the acromion, and the relative locations with respect to the cluster marker of the three scapula landmarks, the *trigonum spinae*, *angulus acromialis*, and *angulus inferior* were pointered before the bench press trials. The local coordinate system of the scapula is created based on these bony landmarks, and the cluster marker is used to reconstruct the translation and rotation of this coordinate system during the trials. Landmark pointering was performed with the participant standing in a neutral position and for the up-, mid-, and down-positions (both with neutral and retracted scapulae) similar to the bench press positions, but while seated upright instead of lying down so that the scapula could be palpated, and landmarks located. Subsequently, the participants had to perform a warm-up protocol. During the measurements, the participants had to perform 21 different bench press techniques (Supplementary Table S2) with three repetitions per technique. A relatively light barbell weight of 16 kg was used, to minimize the injury risk and to ensure that the participant’s movement pattern was not affected by fatigue.

### 2.4 Bench press techniques

**FIGURE 1:**
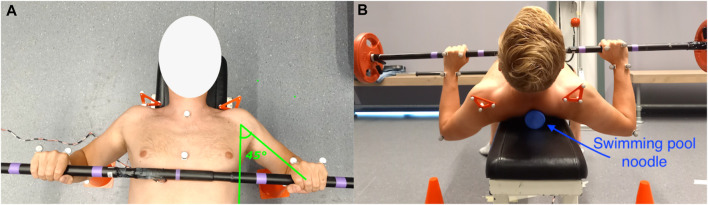
Pictures of the experimental setup. **(A)** shows the purple tape that was placed on the barbell to indicate the grip width and shows an example shoulder abduction angle of 45°. **(B)** shows the orange cones placed beneath participants’ elbows at the desired shoulder abduction angle to monitor if the correct angles were maintained during the bench press. In addition, this picture shows the swimming pool noodle that was placed beneath the spine of the athlete, to release the scapulae from the bench. It must be noted that the participant only lifted his head in **(B)** to show the swimming pool noodle. During the actual measurements, the head was always in contact with the bench.

The performed bench press techniques included combinations of variations in grip width of 1, 1.5 and 2 bi-acromial widths (BAW), shoulder abduction angles of 45, 70° and 90°, and scapula poses including neutral, retracted, and released conditions (Figure 1). When all technique components were combined there were a total of 27 (3 × 3 × 3) techniques. Out of the 27 conditions, 6 were ultimately excluded as these led to unsafe positions (e.g., 90° abduction combined with a small grip width of 1 BAW). The bi-acromial widths of the participants were measured, and the grip width was indicated with tape on the barbell (Figure 1A). A goniometer was used to determine the desired shoulder abduction angle and, in this pose, two cones were placed beneath the elbows (Figure 1B). Participants practiced performing the bench press with the desired shoulder abduction angle beforehand, and the experimenters checked if the elbows were above the cones and provided feedback to the participants to ensure that the correct angles were used during the trials. For the scapula pose, participants were asked to squeeze their shoulder blades together and slightly arch their back for the retracted condition, whereas neutral shoulder blade and back positions were requested for the neutral condition. For the released condition, a swimming pool noodle was placed underneath the spine, to release the scapulae from the bench (Figure 1B) based on the suggestion of Larsen (2008). Instructions for the released condition were the same as for the neutral condition.

### 2.5 Materials

Body segment motions were recorded by 12 infrared cameras from Qualysis (version 2019.3; Qualisys AB, Gothenburg, Sweden) with a sample frequency of 100 Hz. Lateral forces exerted by the hands were measured by a strain gauge force sensor that was integrated in the middle of a barbell with a diameter of 28 mm (Olympic bar). To minimize the effect of barbell bending force on the strain gauges, the bending force was measured before the experiment for a range of grip widths, and corrected for in the post-analysis. In addition, it was ensured that the strain gauges remained on the side of the barbell during the experiment to further minimize the bending force. The forces were recorded with a custom LabView program and measured with a sample frequency of 48 Hz. The motion capture computer and force capture computer were physically connected by a cable, and a pulse signal was used to allow for synchronization of the motion and force data. Qualysis Track Manager software was used to label the marker data.

### 2.6 Data preprocessing

The motion and force data were preprocessed in MATLAB (2021b), The MathWorks, Inc., Natick, Massachusetts, United States). Force data were filtered with a fourth order zero phase lag lowpass Butterworth filter with a cutoff frequency of 6 Hz. Subsequently, the force data were up-sampled by linear interpolation to 100 Hz before synchronization with the motion data. The gravity force component, assumed at 8 kg per hand, and the measured lateral force component were combined into one force vector acting on the center of the hand. For the motion data, small gaps in the marker trajectories were filled by spline interpolation, under the assumption of rigid bodies. Some trials with larger marker gaps had to be excluded. Furthermore, additional body landmark trajectories had to be calculated for the motion data, including the wrist, elbow, and shoulder joint centers. In addition, the scapula landmark trajectories had to be reconstructed based on the acromion cluster marker data. As mentioned, the relative positions of the scapula landmarks in relation to the cluster coordinate system were measured for the up-, mid-, and down-position (100%, 50%, and 0% height) of the bench press. A linear regression was fitted on the x, y, and z coordinates of the landmark coordinates for the three positions, and with this equation the scapula landmarks were reconstructed for each height in the bench press cycle. The motion and force data were synchronized by using the pulse signal, and the data were exported in the correct format for the OpenSim software.

### 2.7 Musculoskeletal modeling

**FIGURE 2:**
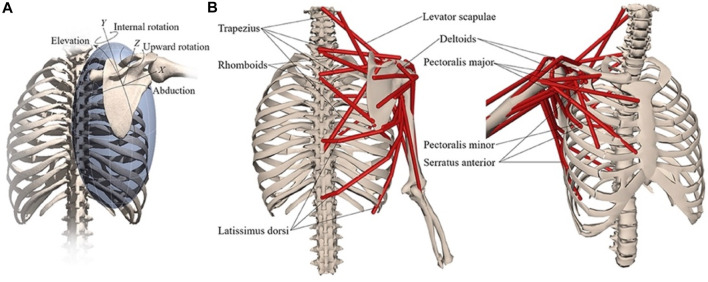
Musculoskeletal model with **(A)** scapular degrees-of-freedom and **(B)** shoulder muscles that control the scapula. Reprinted from Seth et al. (2019).

The musculoskeletal shoulder model from Seth et al. (2019) (Figure 2) was used in the present study, and simulations were performed in the open-source software of OpenSim (version 4.3) (Delp et al., 2007; Seth et al., 2018). The model includes muscle parameters and architecture based on Breteler et al. (1999), with aggregated muscle bundles from Van der Helm (1994) (muscle parameters can be found in Seth et al. (2019); Table 1), combined with a model of scapulothoracic joint kinematics (Seth et al., 2016).

**TABLE 1:** Horizontal (mediolateral) components of the (one) hand forces exerted by the participants on the barbell for each technique condition, as mean ± SD over subjects. Median forces were calculated first for all time-frames in a repetition, subsequently averaged over all repetitions and over all trials of the technique condition, and finally presented as the mean and standard deviations over all participants (positive values indicate forces oriented laterally, while medial forces are negative). Forces are expressed as percentages of the vertical component of the exerted (one) hand force, which is assumed at 78.48 N in the present study.

	Grip width			Scapula pose			Shoulder abduction angle		
Horizontal force [N]	1	1.5	2	N	RT	RL	45°	70°	90°
Median	23.4 ± 63.5	21.2 ± 57.7	29.6 ± 52.8	18.9 ± 59.8	35.7 ± 58.7	15.1 ± 58.1	26.1 ± 61.0	21.6 ± 63.0	22.9 ± 51.1
10th percentile	11.8 ± 68.5	11.7 ± 61.1	22.0 ± 53.7	9.5 ± 63.6	25.5 ± 61.8	5.1 ± 61.5	15.1 ± 65.2	11.5 ± 67.1	14.9 ± 53.0
90th percentile	33.1 ± 58.7	30.7 ± 53.7	38.6 ± 49.9	29.0 ± 55.0	44.2 ± 55.6	25.3 ± 53.5	36.1 ± 57.2	31.6 ± 58.2	32.0 ± 47.5

**FIGURE 3:**
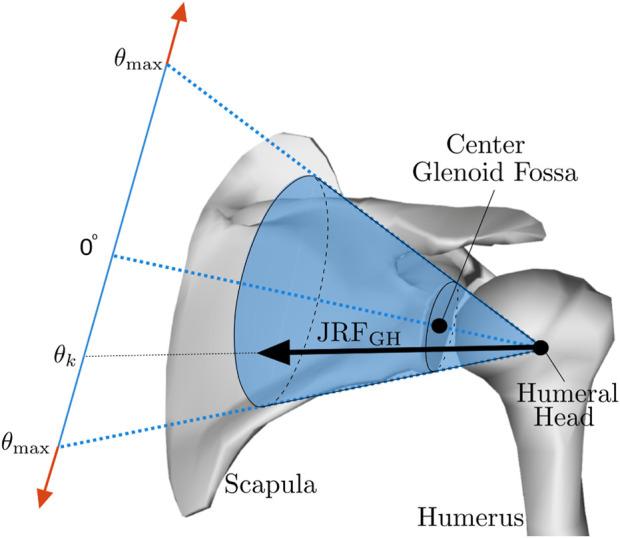
Glenohumeral stability constraint. If the glenohumeral joint reaction force (JRF_GH_) threatens to point outside of the glenoid fossa, indicated by *θ* exceeding *θ*_max_, the humeral head stabilizers will typically be activated to ensure that the reaction force remains directed within the glenoid fossa. Reprinted from Belli et al. (2023).

First, a subject-specific model was created for each participant, by scaling the generic model towards the participant’s dimensions measured in a static trial. For each segment, scaling factors were calculated from the measured bony landmark coordinates. Second, the subject-specific models were fitted to the recorded bench press trials, in a process called Inverse Kinematics (IK), which minimized the squared distances between the experimentally collected marker trajectories and the corresponding virtual markers on the model. Thirdly, activations for the muscle bundles in the model were estimated by means of the “Rapid Muscle Redundancy” (RMR) solver (Belli et al., 2023). During RMR, the joint values, positions and accelerations that result from the inverse kinematic solution and the external force applied to the hand are calculated first, and the minimum required individual muscle activations are estimated from these quantities by solving an optimization problem, where muscle effort is minimized under a few constraints. The RMR solver is a new method that, as opposed to the widely used static optimization method in OpenSim, accounts for the stability of the glenohumeral joint, and can activate the humeral head stabilizers (e.g., the rotator cuff muscles) if the direction of glenohumeral reaction force threatens to point outside of the glenoid fossa (Figure 3). In addition, the RMR solver includes passive muscle forces and guarantees physiological consistency of the activations between adjacent time instants (Belli et al., 2023). To get an indication of the instability in the glenohumeral joint for the different bench press techniques, the activities of the rotator cuff muscles, which are important humeral head stabilizers, are reported. In addition, the activities of the pectoralis major muscles are reported, since these typically represent the target muscles of the bench press exercise. Finally, joint reaction forces in the glenohumeral and acromioclavicular joints were estimated based on the measured external force and the computed muscle forces. The joint reaction forces were split into a pure compression component, pointing from the center of the humeral head to the center of the glenoid, and shear components in the anterior-posterior and superior-inferior directions. For the glenohumeral joint, compression and shear force components are defined based on the local coordinate system of the scapula with the origin in the center of the glenoid. The glenohumeral shear force component would be zero if the reaction force is directed exactly at the center of the glenoid fossa, and larger than zero if the reaction force would be directed further away from the glenoid center. For the acromioclavicular joint, compression and shear force components are defined based on the local coordinate system of the clavicle with the origin in the acromioclavicular joint. These components were reported alongside the total reaction forces in the glenohumeral and acromioclavicular joints.

### 2.8 Statistical analyses

Joint reaction force timeseries were normalized to percentages of the bench press phase, with 0% representing the start of the cycle with the barbell up, 50% representing the lowest point of the barbell, and 100% representing the end with the barbell at the highest point again. Statistical non-parametric mapping from the spm1d package (Pataky, 2012) (available from https://spm1d.org/) in Python (Python Software Foundation, Python Language Reference, version 3.3, available at http://www.python.org) was used to identify the presence and phase of significant differences between the time-series. A hierarchical two-level random effects analysis was used. At the first level, within-subject effects were estimated by multiple linear regression. The predictors grip width, abduction angle and scapula pose, all categorical variables (factors) containing three levels (in the regression analysis resulting in two dummy variables per factor), and the two-way interactions (12 terms in total) and three-way interactions (8 terms in total) between these factors were included to predict each joint reaction force component. This resulted in beta-coefficient continuums for all factors and interaction terms for each participant. At the second level, a statistical non-parametric mapping one-sample *t*-test was conducted on the beta continuums of all participants together to identify if and when during the bench press cycle the continuum was significantly different from zero (*p* < 0.05), indicating a significant difference between conditions. If none (or only a few) of the three-way interactions appeared significant, these terms were excluded from the model and the two steps were performed again. Subsequently, the two-way interactions were assessed for significance and excluded otherwise. If none or only a few of the interaction terms were significant, only the effects of the main factors (corrected for the effect of the other factors) were reported in the results. If interactions were present, the statistical analysis was conducted separately for each level of the relevant factors.

## 3 Results

Twelve percent of the trials had to be excluded due to missing markers or a missing lateral barbell force. Being an important component of the input of the OpenSim musculoskeletal model, Table 1 presents the descriptive statistics of the mediolateral component of the hand forces exerted by the participants on the barbell, expressed as percentage of the vertical component (8 kg ≈ 78.48N). It can be observed that mean forces are typically directed in the lateral direction and are somewhat larger for the wide grip (2BAW) condition and the retracted scapulae condition. However, as indicated by the large standard deviations, between-individual differences in mean mediolateral forces are very large.

**FIGURE 4:**
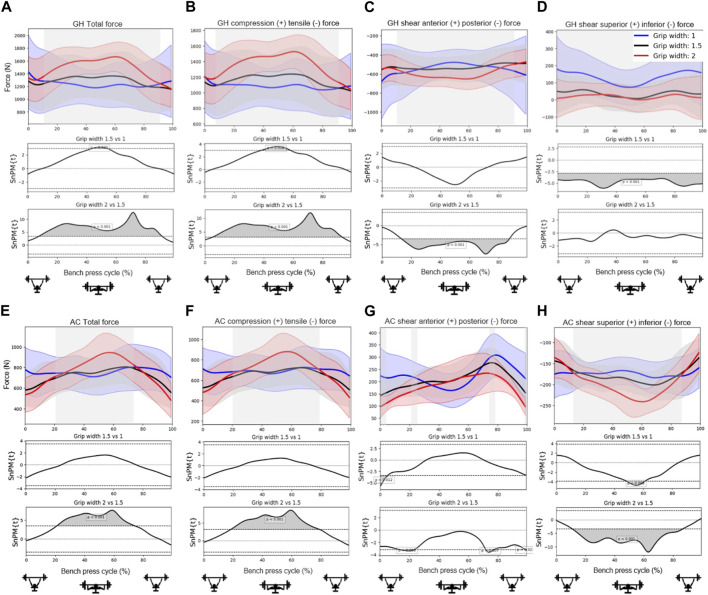
Effects of *grip width* on the **(A)** total glenohumeral reaction force, **(B)** glenohumeral compression force, **(C)** glenohumeral anterior shear force component, **(D)** glenohumeral superior shear force component, **(E)** total acromioclavicular reaction force, **(F)** acromioclavicular compression force, **(G)** acromioclavicular anterior shear force component, and **(H)** acromioclavicular superior shear force component. Top subplots show the mean and between-subject variation (shaded) in forces for grip widths 1 (blue), 1.5 (black) and 2 (red) times the bi-acromial-width during a bench press cycle with a total barbell mass of 16 kg. The middle and bottom subplots represent the results from statistical non-parametric mapping (SnPM) indicating if and when during the bench press cycle there is a significant difference (grey) on the outcome parameter between grip widths.

**FIGURE 5:**
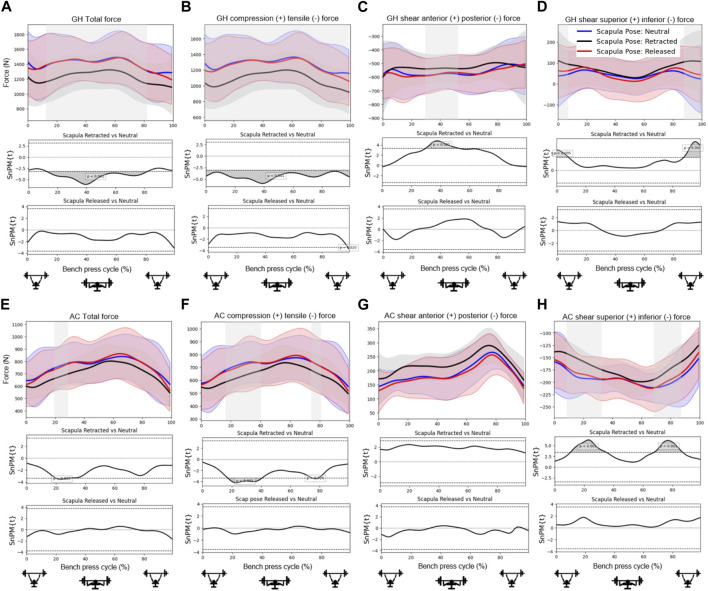
Effects of *scapula pose* on the **(A)** total glenohumeral reaction force, **(B)** glenohumeral compression force, **(C)** glenohumeral anterior shear force component, **(D)** glenohumeral superior shear force component, **(E)** total acromioclavicular reaction force, **(F)** acromioclavicular compression force, **(G)** acromioclavicular anterior shear force component, and **(H)** acromioclavicular superior shear force component. Top subplots show the mean and between-subject variation (shaded) in forces for the scapula poses Neutral (blue), Retracted (black) and Released (red) during a bench press cycle with a total barbell mass of 16 kg. The middle and bottom subplots represent the results from statistical non-parametric mapping (SnPM) which shows if and when during the bench press cycle there is a significant difference (grey) on the outcome parameter between scapula poses.

**FIGURE 6:**
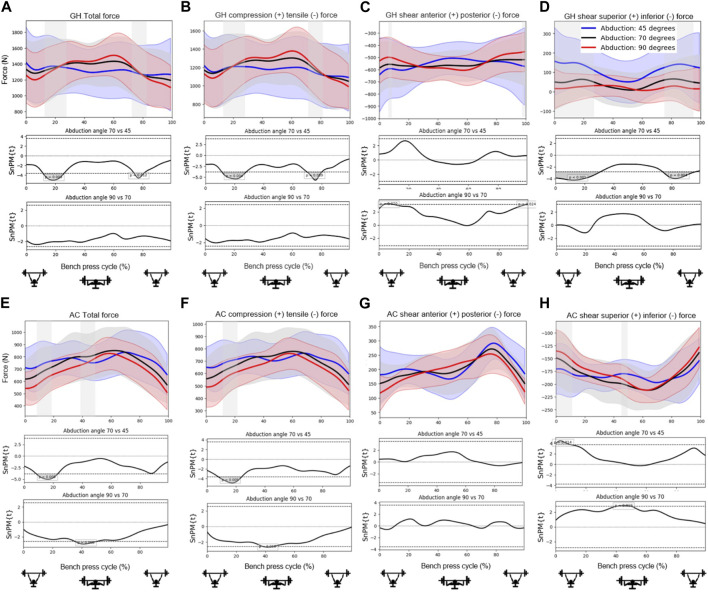
Effects of *shoulder abduction angle* on the **(A)** total glenohumeral reaction force, **(B)** glenohumeral compression force, **(C)** glenohumeral anterior shear force component, **(D)** glenohumeral superior shear force component, **(E)** total acromioclavicular reaction force, **(F)** acromioclavicular compression force, **(G)** acromioclavicular anterior shear force component, and **(H)** acromioclavicular superior shear force component. Top subplots show the mean and between-subject variation (shaded) in forces for the shoulder abduction angles 45 (blue), 70 (black) and 90 (red) degrees during a bench press cycle with a total barbell mass of 16 kg. The middle and bottom subplots represent the results from statistical non-parametric mapping (SnPM) which shows if and when during the bench press cycle there is a significant difference (grey) on the outcome parameter between shoulder abduction angles.

Figure 4, Figure 5, Figure 6 show the mean total force and the compression and shear force components in the glenohumeral and acromioclavicular joints for different grip widths, scapula poses and abduction angles during a bench press cycle. Figure 4 shows that larger grip widths significantly increased the total glenohumeral reaction force, glenohumeral compression, the glenohumeral posterior shear force component, total acromioclavicular reaction force, acromioclavicular compression, and the acromioclavicular inferior shear force component during the main part of the bench press cycle. These forces especially showed a significant increase when the grip width was widened from 1.5 to 2 bi-acromial widths (BAW). Furthermore, a small grip width of 1 BAW increased the glenohumeral superior shear force component compared to a grip width of 1.5 BAW (Figure 4D). Figure 5 shows that scapula retraction as compared to a neutral scapula position significantly decreased the total glenohumeral reaction force and glenohumeral compression during the main part of the bench press cycle (Figures 5A,B). Around the lowest point in the bench press cycle, retraction decreased the glenohumeral posterior shear force component (Figure 5C). At the very start and end of the bench press cycle, retraction increased the glenohumeral superior shear force component (Figure 5D). In addition, retraction decreased acromioclavicular compression and the inferior shear force component during the middle of the descending and ascending phases (Figures 5F,H). No differences in joint reaction forces between released and neutral scapulae were observed. Figure 6 shows that a small shoulder abduction angle of 45° increased the glenohumeral superior shear force component compared to 70° shoulder abduction during the descending and ascending phases of the bench press. Furthermore, larger shoulder abduction angles increased total glenohumeral reaction forces and glenohumeral compression force components, and decreased glenohumeral superior shear force components, total acromioclavicular forces, acromioclavicular compression force components and acromioclavicular inferior shear force components. However, these differences were only present during very short parts of the bench press cycle.

**FIGURE 7:**
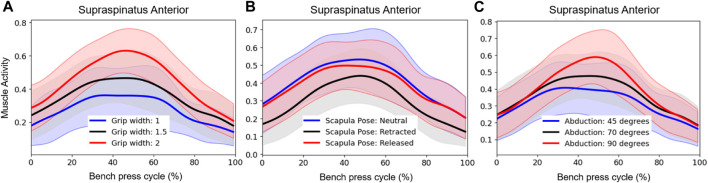
Effect of **(A)** grip width, **(B)** scapula pose, and **(C)** shoulder abduction angle on Supraspinatus Anterior activity. Subplots show the mean and between-subject variation (shaded) in activities for the different technique conditions (blue, red, black) during a bench press cycle with a total barbell mass of 16 kg. Muscle activities are expressed relative to a maximum activity of 1.

It can be observed from Table 2 that peak rotator cuff activities were always smaller for retracted scapulae, meaning that less rotator cuff activity was required during this technique to maintain the humeral head within the glenoid. In addition, Table 2 shows that the supraspinatus and subscapularis activities were smaller for smaller grip widths. Differences were largest for the supraspinatus anterior. To gain more insight into the course of the activity from this muscle during different bench press techniques, we plotted the supraspinatus anterior activity time series in Figure 7. This figure shows that supraspinatus anterior activity peaks around the lowest point in the bench press, and that scapula retraction and a small grip width reduces the activity throughout the whole movement. Around the peak, smaller shoulder abduction angles also decrease the supraspinatus anterior activity. Furthermore, Table 2 shows that the lowest pectoralis major activities were found for small grips, retracted scapulae, and small shoulder abduction angles.

**TABLE 2:** Peak muscle activities for the rotator cuff and pectoralis major, as mean ± SD over subjects per bench press technique component. Peak muscle activities were calculated first for all time-frames in a repetition, subsequently averaged over all repetitions and over all trials of the technique component. Muscle activities are expressed relative to a maximum activity of 1. Green cells indicate the lowest peak muscle activity per technique component for each modeled part of the rotator cuff and pectoralis major muscles.

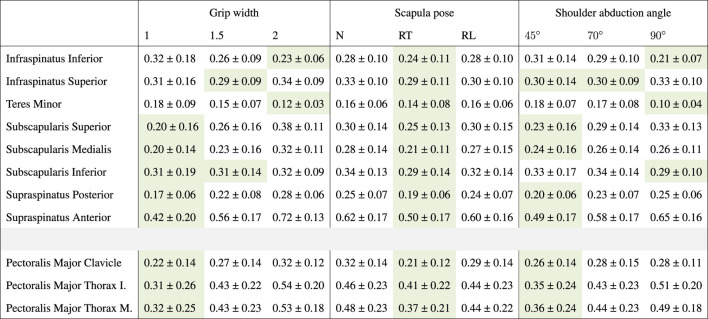

## 4 Discussion

The aim of the present study was to investigate the effect of bench press technique on joint reaction forces in the glenohumeral and acromioclavicular joints during the bench press, as bench press techniques with lower joint loads may decrease the risk for particular injuries. Results showed that especially grip width and scapula pose largely affected the size and direction of reaction forces in the glenohumeral and acromioclavicular joints. Narrower grips and retracted scapulae significantly decreased the total glenohumeral reaction force, resulting in lower glenohumeral compression, glenohumeral posterior shear force components, and decreased the total acromioclavicular reaction force, resulting in lower acromioclavicular compression and inferior shear force components.

The larger reaction forces in the glenohumeral and acromioclavicular joints for wider grips that were found in the present study may be explained by the larger moment arms of the external (barbell) forces at the hands with respect to the joint centers. These result in larger joint moments, which is in line with the larger shoulder moments for wider grips found by Larsen (2008) and Mausehund et al. (2022). These larger moments subsequently require more muscle activity, as was shown by the larger pectoralis major activities for wider grips in Table 2, which leads to larger reaction forces in the shoulder joints. While Larsen (2008) and Mausehund et al. (2022) recommended wide grips for more muscle adaptation and higher weights to be lifted, our results also indicate that wide grips may increase the injury risk as these lead to higher joint reaction forces. In particular, the higher compression forces that were found to act on the distal clavicle for wide grip widths of 2 BAW support the theory of Green and Comfort (2007) that wider grips may increase the risk for distal clavicular osteolysis. These results show that there is a trade-off between performance and injury risk when choosing the bench press grip width, which must be considered carefully by athletes and coaches.

Moreover, it has been suggested that wide grips may lead to glenohumeral instability problems. In line with this theory, it was found in the present study that glenohumeral shear force components were always directed posteriorly during the bench press (Figure 4C; Figure 5C; Figure 6C) and were larger for wider grips (Figure 4C). In addition, the rotator cuff muscles subscapularis and supraspinatus, which may oppose the glenohumeral posterior shear force component, showed larger activities for wider grips (Table 2; Figure 7A). Green and Comfort (2007) and Durall et al. (2001) proposed that bench pressing places high strain on the inferior glenohumeral ligament and anterior glenohumeral ligament. In the present study, the rotator cuff muscles were able to maintain the humeral head in the glenoid. However, if heavier weights are used, it is possible that the anterior rotator cuff muscles are unable to restrain the humeral head, and the inferior glenohumeral ligament and anterior glenohumeral ligament are required to oppose the posterior shear force component. Herewith, our results do provide support for the theory of Green and Comfort (2007) and Durall et al. (2001) that bench pressing with wide grips may increase the risk for glenohumeral anterior instability and rotator cuff injuries.

Scapula retraction was found to decrease both compression and posterior shear force components in the glenohumeral joint. In addition, all rotator cuff muscle activities were lower for the retracted condition (Table 2), indicating that retraction may have helped to keep the humeral head relatively more centered in the glenoid fossa, placing less load on the glenohumeral stabilizers. Figure 7 showed that retraction decreased the supraspinatus anterior muscle activity throughout the whole movement cycle. The observed differences in rotator cuff loads seem important, especially considering that results showed large glenohumeral posterior shear force components in general during the bench press, highlighting that the demand on the stabilizers is already high. Therewith, these results support the guidelines of the NSCM, stating that continuous scapula retraction should be performed during the bench press (Graham, 2003), as it may decrease the risk for glenohumeral instability and rotator cuff problems. Regarding the performance, we did observe lower pectoralis major activities for retracted shoulders (Table 2). However, García-Ramos et al. (2018) showed that there was no significant difference in the 1RM between a retracted and more neutral scapula condition during the bench press.

Furthermore, no evidence was found in the present study to support the theory of (Larsen, 2008) that releasing the scapulae with a pool noodle would help to remain the humeral head more centered in the glenoid. In fact, no differences in reaction forces between the released and neutral scapulae conditions were observed at all. This suggests that perhaps scapula mobility is not restricted by the bench as much as expected, or that it is not as free as expected when released.

Overall, shoulder abduction angles were shown to have a smaller effect on the joint reaction forces than grip width and scapula pose. The most significant finding showed that a small abduction angle of 45° led to larger glenohumeral superior shear force components during the start and end of the bench press (Figure 6D). This is in line with the larger glenohumeral shear force components that were observed for a narrow grip of 1 BAW (Figure 4D). This suggests that for athletes experiencing problems or pain in the area above the glenohumeral joint, for instance experiencing subacromial pain syndrome, it might be better to avoid small shoulder abduction angles of 45° and narrow grip widths of 1 BAW.

Table 1 showed that on average, lateral components of the forces exerted by the hands on the barbell were larger for a wider grip of 2 BAW compared to narrower grips of 1 and 1.5 BAW, which is in line with previous literature (Larsen et al., 2021; Mausehund et al., 2022). In addition, the present study found that the lateral components of the exerted hand forces were larger for retracted scapulae compared to neutral and released scapulae (Table 1). However, most strikingly, individual differences in exerted mediolateral forces were found to be very large (Table 1). The positive median values ranging from about 15% to 35% of the vertical component of the exerted hand force indicate that in general, the exerted hand forces were directed laterally, but the large standard deviations up to 64% indicate that some participants exerted much larger forces in the lateral direction, or exerted forces in the medial direction. In line with this, varying values ranging from about 10% medially directed forces to about 40% laterally directed forces (as a percentage of the vertical force) have previously been found during the bench press (Duffey and Challis, 2011; Larsen et al., 2021; Mausehund et al., 2022). Although part of the variation found in the literature may be explained by different grip widths, the results of the present study show that the grip width cannot explain all variation in the observed medial or lateral directed exerted hand forces, as these still varied considerably within the technique conditions (Table 1).

In theory, it could be expected that for grips wider than 1 BAW, exerting laterally directed hand forces will direct the resultant reaction force at the hands towards the shoulder joint, decreasing the shoulder moment arms, which seems most efficient and may help to decrease shoulder reaction forces. To investigate the effect of the level of more medially or laterally directed exerted hand forces at the barbell on shoulder reaction forces, we conducted an additional simulation, wherein we systematically changed the direction (and thus also the size) of the exerted hand forces varying from 50% medial to 200% lateral directed forces (in % of the vertical force) to the kinematics of one example trial (P7, Neutral scapulae, 70° abduction, 1.5 BAW). The Supplementary Figure S1 shows the resulting (Euclidian norm of the) mean reaction forces in the glenohumeral and acromioclavicular joint for each of the simulated mediolateral forces. In line with our expectations, it can be observed that except for the anterior-posterior shear force components, the forces of all joint reaction components are smaller when barbell forces are exerted in the lateral direction. For the glenohumeral joint, the lowest reaction forces were typically found for laterally directed exerted forces of 50–100% of the vertical exerted force component (still fixed at 78.48N), whereas for the acromioclavicular joint, the lowest joint reaction forces were typically found for laterally directed exerted forces of 100–150% of the vertical exerted force component. It must be noted that this is a simulation with ‘fake’ mediolateral components of the exerted forces, only applied to one example trial, and therefore these percentages cannot be generalized. Nevertheless, these results point out that mediolaterally directed exerted hand forces at the barbell have a large effect on glenohumeral and acromioclavicular reaction forces and may influence the injury risk.

Larsen et al. (2021) proposed that the mediolateral exerted force variation between participants might be due to different experience levels, with more experience leading to a more efficient technique. When comparing the mean mediolateral exerted force components between the participants in the present study (Supplementary Table S3), it is striking that two participants (P2 and P10) exerted large medially directed forces, which seems inefficient and could increase glenohumeral and acromioclavicular reaction forces (Supplementary Figure S1). Interestingly, these appeared to be the participants with the least bench press experience, both with 3 years of experience *versus* a mean and SD of 8 ± 4 years of experience for the other participants. These observed inter-individual differences in mediolaterally directed exerted hand forces at the barbell and corresponding differences in shoulder reaction forces, potentially related to the experience level, highlight that it would be interesting to explore the use of instrumented barbells with feedback on the exerted force direction in the gym to optimize performance and decrease injury risk during the bench press.

This study has some strengths and limitations. One of the strengths is that this is, to our knowledge, the first study that applied a musculoskeletal model and estimated shoulder joint reaction forces during multiple bench press techniques. Moreover, another strength of this study is that the results are directly applicable to strength training practice and can be used to develop safe bench press training guidelines in collaboration with coaches and clinicians. However, limitations also apply to this study. Firstly, although we infer on how the results of the present study can be related to reported bench press injuries, it must be noted that this study design does not allow for the identification of actual injury risks since this is not a prospective epidemiological study. Secondly, it is important to realize that musculoskeletal models will always find a minimal solution of activations required to produce the measured kinematics and external forces, and participants will not always activate their muscles in line with the artificial cost function. In addition, musculoskeletal models are based on several assumptions regarding muscle parameters, geometries, and joint kinematics, and in this case also aggregated muscles (Seth et al., 2011). In addition, ligaments are not included in the current model. These limitations may have led to deviations from actual muscle and joint forces. However, although validation of musculoskeletal models is complex since it is difficult to measure muscle and joint forces *in vivo*, Seth et al. (2019) found agreement between simulated and EMG-measured muscle activations during multiple tasks for the model used in the present study. Furthermore, a study with an instrumented prothesis showed that glenohumeral reaction forces estimated by the Delft Shoulder and Elbow Model (DSEM), which formed the basis for the model applied in the present study, showed reasonable compatibility with measured reaction forces for multiple motions (Nikooyan et al., 2010). Thirdly, although the effect of bending strain on the barbell force measurements was minimized by subtracting bending forces that where measured beforehand in a static trial, it is possible that different bending forces were present during the dynamic measurements due to the effect of inertial forces. Finally, the vertical external force component could not be measurement by the instrumented barbell in the present study and was assumed to be equal to the gravitational force of the total load and evenly distributed over the two hands. Depending on the vertical accelerations of the barbell, and vertical components of the exerted hand forces in the present study, this may have led to slight underestimations of the vertical barbell force component, which was found to be 113–115% of the total load for submaximal and maximal lifts in the study of Duffey and Challis (2011). However, previous research pointed out that especially the horizontal force component is important to measure as it has a major effect on the joint moments during the bench press (Mausehund et al., 2022).

It can be concluded that the bench press technique, especially the grip width and scapula pose, influences the size and direction of reaction forces in the glenohumeral and acromioclavicular joints. In general, using a medium or narrow grip width below 1.5 BAW and retracting the scapulae decreases compression and shear force components in the glenohumeral and acromioclavicular joints. This may decrease the risk for certain bench press injuries. In particular, grip widths of <1.5 BAW decrease the compression force on the distal clavicle and may decrease the risk for distal clavicular osteolysis. Moreover, retracting the scapulae, as well as using a grip width of <1.5 BAW, decreases glenohumeral posterior shear force components and may decrease the risk for glenohumeral posterior instability and rotator cuff problems. The observed inter-individual differences in mediolateral direction of the exerted hand forces applied to the barbell and the corresponding large effect on joint reaction forces observed during a musculoskeletal simulation indicate that these exerted forces at the barbell are also an important technical component for athletes to focus on to make the bench press safer and more efficient. Results of this study can contribute to improving safe bench press training guidelines for athletes in collaboration with coaches and clinicians.

## Data Availability

The raw data supporting the conclusion of this article will be made available by the authors, without undue reservation.
