# Supplementary material for: Effects of bench press technique variations on musculoskeletal shoulder loads and potential injury risk
Source: Front Physiol. 2024 Jun 21;15:1393235. doi: 10.3389/fphys.2024.1393235 (PMC11224528; doi:10.3389/fphys.2024.1393235)
Supplement: Supplementary file 1 [file DataSheet1.PDF]

frontiers

Supplementary Material

1 TABLES

Table S1. Anatomical locations of experimental markers per body segment.

Segment	Anatomical location
Thorax	Incisura Jugularis<br>Processus Xiphoideus<br>Cervical Vertebrae 7<br>Thoracic Vertebrae 6<br>Thoracic Vertebrae 6
Scapula	Acromion-cluster marker (3 markers)<br>to track the translation and orientation<br>of the pointered scapula landmarks below.<br>Trigonum Spinae (pointered)<br>Angulus Acromialis (pointered)<br>Angulus Inferior (pointered)
Humerus	Epicondylus Medialis<br>Epicondylus Lateralis
Lower Arm	Ulnar Styloid<br>Radial Styloid
Hand	2nd Metacarpal Head (MH2)<br>5th Metacarpal Head (MH5)

Table S2. All bench press techniques with different combinations of scapula poses, shoulder abduction angles, and grip widths.

Technique code	Scapula pose	Shoulder abduction angle [°]	Grip width (in bi-acromial widths)
1	Neutral	45	1
2	Neutral	45	1.5
3	Neutral	70	1
4	Neutral	70	1.5
5	Neutral	70	2
6	Neutral	90	1.5
7	Neutral	90	2
8	Retracted	45	1
9	Retracted	45	1.5
10	Retracted	70	1
11	Retracted	70	1.5
12	Retracted	70	2
13	Retracted	90	1.5
14	Retracted	90	2
15	Released	45	1
16	Released	45	1.5
17	Released	70	1
18	Released	70	1.5
19	Released	70	2
20	Released	90	1.5
21	Released	90	2

Table S3. Mean horizontal barbell forces for all participants expressed in Newton and as percentage of the vertical force. Positive values/variations indicate lateral forces, negative values/variation indicate medial forces.

	Mean horizontal force [N]	Mean horizontal force [% vertical force]
P1	71.8	91.5
P2	-49.0	-62.4
P3	64.7	82.4
P4	20.1	25.6
P5	3.2	4.1
P6	90.9	115.8
P7	11.2	14.3
P8	-15.2	-19.4
P9	17.1	21.8
P10	-34.4	-43.8

2 FIGURES

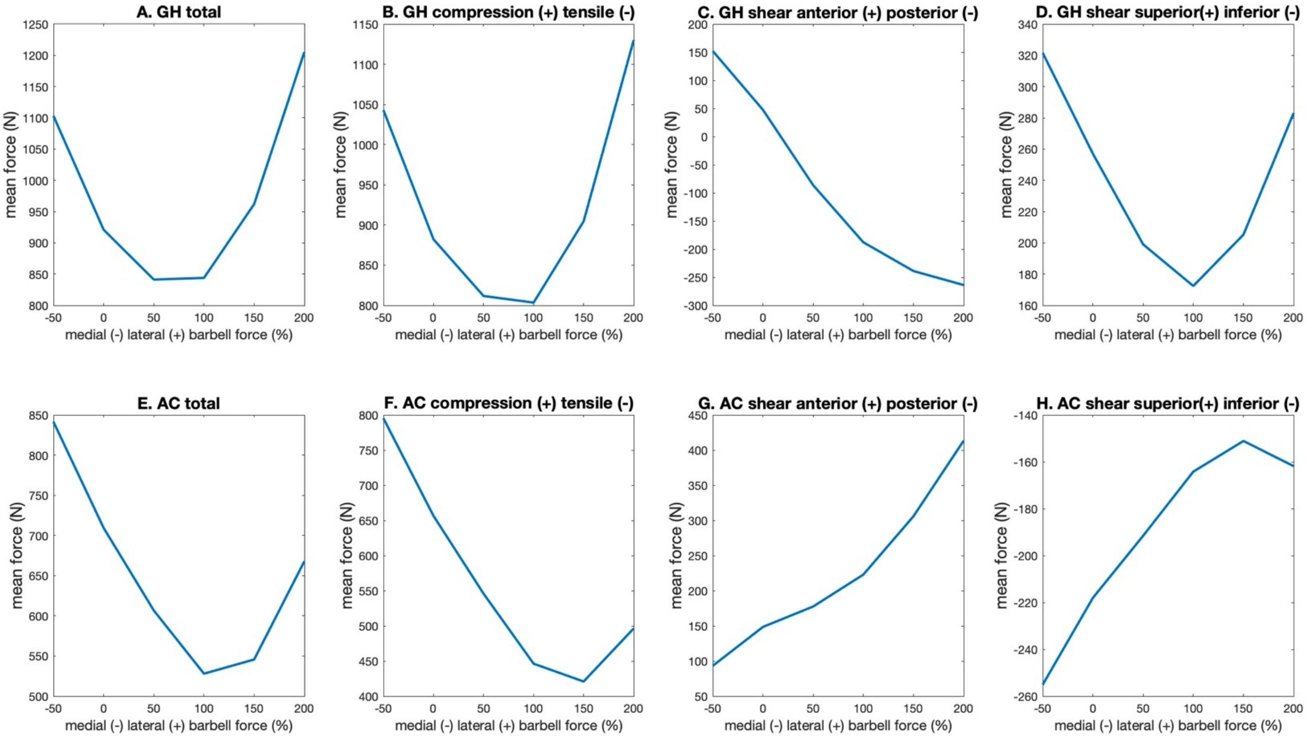

Figure S1. Mean reaction forces in the glenohumeral and acromioclavicular joints for simulated mediolateral barbell forces during one example bench press trial. Mean joint reaction forces were first calculated from the timeseries of each repetition and subsequently averaged over the multiple repetitions. Simulated mediolateral forces are expressed as percentage of the vertical force.
